# Emergent or urgent hybrid repair for complex thoracoabdominal aortic aneurysms: single-centre experience

**DOI:** 10.3389/fcvm.2026.1949749

**Published:** 2026-10-28

**Authors:** Linyao Zhu, Yuhan Qi, Jiarong Wang, Chengxin Weng, Hankui Hu, Qiang Guo, Tiehao Wang, Xiyang Chen, Yi Yang, Ding Yuan, Bin Huang, Jichun Zhao

**Affiliations:** 1 Division of Vascular Surgery, Department of General Surgery, West China Hospital, Sichuan University, Chengdu, China; 2 West China School of Medicine, Sichuan University, Chengdu, China; 3 Department of Vascular Surgery, The First People’s Hospital of Yunnan Province, Kunming, China

**Keywords:** emergency medicine, hybrid, single arm clinical study, TAAA=thoracoabdominal aortic aneurysm, urgent

## Abstract

**Objective:**

Emergent or urgent repair of thoracoabdominal aortic aneurysms (TAAA) always represent a major challenge due to high mortality and complex reconstruction of visceral arteries. We aimed to evaluate the short- and long-term outcomes of patients undergoing emergent or urgent hybrid TAAA repair under a standardized institutional perioperative protocol.

**Methods:**

This study represents a retrospective cohort analysis of data derived from a prospectively maintained institutional database at West China Hospital. All consecutive patients who underwent emergent or urgent hybrid repair for TAAA between January 2013 and February 2025 were included.

**Results:**

The cohort comprised 38 TAAA patients (31 male, with a median age of 50.5 years) who underwent emergent/urgent hybrid repair. The median aneurysm length was 200.9 mm (IQR 154.0-320.0), with a mean maximum diameter was 70.5 ± 17.8 mm. Intraoperatively, 4 visceral arteries (IQR 4-5) were reconstructed. Median operative time was 415 min (IQR 360-504), with median ICU and postoperative hospital stays of 96.0 h (IQR 46.5-203.5) and 17 days (IQR 11-24), respectively. No myocardial infarction or hepatic failure occurred. Postoperative complications included pulmonary infection (*n* = 4, 10.5%), acute kidney injury (*n* = 2, 5.3%), and spinal cord ischemia (*n* = 5, 13.2%). Thirty-day mortality was 10.5% (*n* = 4). The survival rates at 12 and 60 months postoperatively were 86.8% (95% CI 76.7–98.3) and 83.0% (95% CI 71.7–96.6). The free of reintervention rates at 12 and 60 months postoperatively were 90.5% (95% CI 80.8–100.0) and 76.4% (95% CI 61.1–95.7).

**Conclusion:**

In this selected cohort managed with a standardized perioperative protocol, emergent and urgent hybrid TAAA repair was feasible, with acceptable perioperative mortality and durable mid- to long-term survival. However, the occurrence of spinal cord ischemia represents a substantial and clinically significant trade-off of this approach in the emergency setting. Moreover, these favorable survival outcomes should be interpreted in the context of the cohort's relatively young age and favorable baseline risk profile. These findings emphasize that survival benefit is counterbalanced by significant neurological morbidity, highlighting the need for refined spinal protection protocols in urgent cases and for comparative studies to validate the specific efficacy of this standardized protocol.

## Introduction

Thoracoabdominal aortic aneurysm (TAAA) repair represents one of the most technically demanding procedures in contemporary vascular surgery, associated with high perioperative morbidity and mortality rates (1, 2). In the early times, open surgical repair has been established as the gold standard for TAAA intervention (3). However, this approach necessitates extensive thoracoabdominal exposure and single-lung ventilation, rendering it potentially unsuitable for high-risk patient populations, particularly those with advanced age or significant comorbidities (4, 5). The evolution of endovascular techniques has revolutionized TAAA treatment paradigms, and the advent of fenestrated and branched stent-graft technology enabled totally endovascular repair of TAAA with promising short-term outcomes (6, 7).

In cases of ruptured or impending rupture TAAA, emergent or urgent TAAA repair is required. However, in such critical scenarios, the utilization of custom-made fenestrated or branched stent grafts is often precluded due to time constraints. While a few specialized centres reported the use of physician-modified off-the-shelf endografts, long-term follow-up data revealed a relatively high incidence of visceral artery-related complications. Furthermore, the complex anatomical alterations associated with ruptured or impending rupture TAAA pose substantial technical challenges for totally endovascular repair. Consequently, the benefits of emergent totally endovascular repair are limited, necessitating careful consideration of alternative therapeutic strategies in these high-risk scenarios.

Hybrid repair was initially designed to combine the privilege of both open and endovascular procedures (8), by first performing retrograde visceral artery debranching with hand-made anastomosis and followed by implantation of standard stent graft to seal the aneurysm sac. Given its reduced dependency on complex stent graft configurations, hybrid repair emerged as a feasible option in emergent clinical scenarios. The pioneering experience of emergent TAAA hybrid repair was first summarized by Dr. M. Keese and colleagues from University Hospital Frankfurt (9), with the advantages of immediate availability and the ability to achieve precise visceral artery reconstruction without the necessity for thoracotomy, transitioning from high supraceliac or thoracic aortic cross-clamping to lower infrarenal aortic or iliac clamping for inflow reconstruction. Nevertheless, despite these procedural benefits, emergent hybrid repair was still associated with high rates of postoperative mortality and morbidity (9).

To address the procedural and hemodynamic challenges of emergent hybrid TAAA repair, a standardized perioperative protocol was established at our institution in 2013. The aim of this study is to describe our single-centre experience and evaluate the short- and long-term clinical outcomes of consecutive patients undergoing emergency or urgent hybrid TAAA repair managed under this standardized clinical pathway.

## Methods

### Study design, settings and ethics

This study is a retrospective single-arm cohort study collected from a prospective database. The study has been approved by the hospital's ethics committee. Since the patient's medical record data were collected anonymously, informed consent was not required. This study complies with the enhanced reporting statement (STROBE) standards for observational epidemiological research reports (as shown in the checklist in Supplementary Table S1).

### Patients

All TAAA patients who underwent emergent or urgent hybrid repair between January 2013 and February 2025 were included in the study. The indication for emergent repair included one of the following criteria: 1) hemodynamically unstable, with mean arterial pressure <90 mmHg; 2) CT scan suggested any signs of ruptured TAAA (the presence of retroperitoneal, or intraperitoneal hematoma, or intrathoracic cavity hematoma adjacent to the aneurysm sac). Urgent TAAA repair were performed if the TAAA was considered impending rupture, which defined as those who had acute onset or exacerbation of abdominal or back pain, with at least one of the following CTA findings: draped aorta sign, well-defined peripheral crescent of increased attenuation within the thrombus of a large TAAA, focal discontinuity in circumferential wall calcifications, collection of fluid around aneurysm sac. Patients were excluded if they were found to have disturbance of consciousness or received cardio-pulmonary resuscitation before the operation.

### Technical aspects of hybrid repair

All emergent or urgent hybrid repairs were performed in the hybrid operation room in one stage. In our cohort of 38 emergency or urgent patients, 1 patient (2.6%) presented with true aortic rupture, defined as acute hemodynamic collapse with a mean arterial pressure (MAP) below 90 mmHg and active extravasation with a large retroperitoneal hematoma on emergent CTA. The remaining 37 patients (97.4%) presented with impending rupture, defined as acute intractable back or abdominal pain accompanied by high-risk CTA instability signs, including a hyperdense crescent sign within the thrombus, draped aorta sign, focal aortic wall discontinuity, or localized periaortic stranding without free systemic rupture.

In the hybrid operating suite, a compliant aortic occlusion balloon (Reliant, Medtronic) was routinely prepared and kept on standby via a contralateral femoral sheath. For the single patient with true rupture and hemodynamic shock, an immediate hybrid protocol was executed. Under simultaneous fluoroscopic guidance and open surgical preparation, a proximal thoracic stent-graft was rapidly deployed via the transfemoral route to achieve prompt hemodynamic stabilization and seal the rupture, followed immediately by open retrograde visceral debranching and completion of distal relining within the same operative session. In hemodynamically stable patients with impending rupture (*n* = 37), standard sequential hybrid repair was performed, consisting of open visceral debranching under controlled permissive normotension (MAP 90–100 mmHg) with cell salvage, followed by endovascular completion. A representative preoperative three-dimensional vascular reconstruction imaging is presented in the Figure 1A. All patients adopted transperitoneal approach through a midline incision to expose abdominal aorta, visceral arteries and bilateral iliac arteries. Abdominal aorta or iliac arteries were often chosen as the common source of inflow, and sometimes bifurcated artificial graft was adopted if necessary. Celiac trunk was dissected through lesser omental sac, the root of superior mesenteric artery (SMA) was dissected cranially to bilateral renal arteries through same approach. During exposure of visceral arteries, a multi-vessel debranching graft was made (details shown in the optimized protocol). The main body of multi-vessel debranching graft was anastomosed end-to-side to the inflow artery, then bilateral renal arteries were reconstructed by end-to-end anastomosis preferably. SMA was revascularized end-to-side at the root, and the celiac branch was tunnelled beneath the pancreas and anastomosed end-to-side at the bifurcation or common hepatic artery. The retrograde debranching anastomosis was shown in Figure 1B.

**Figure 1:**
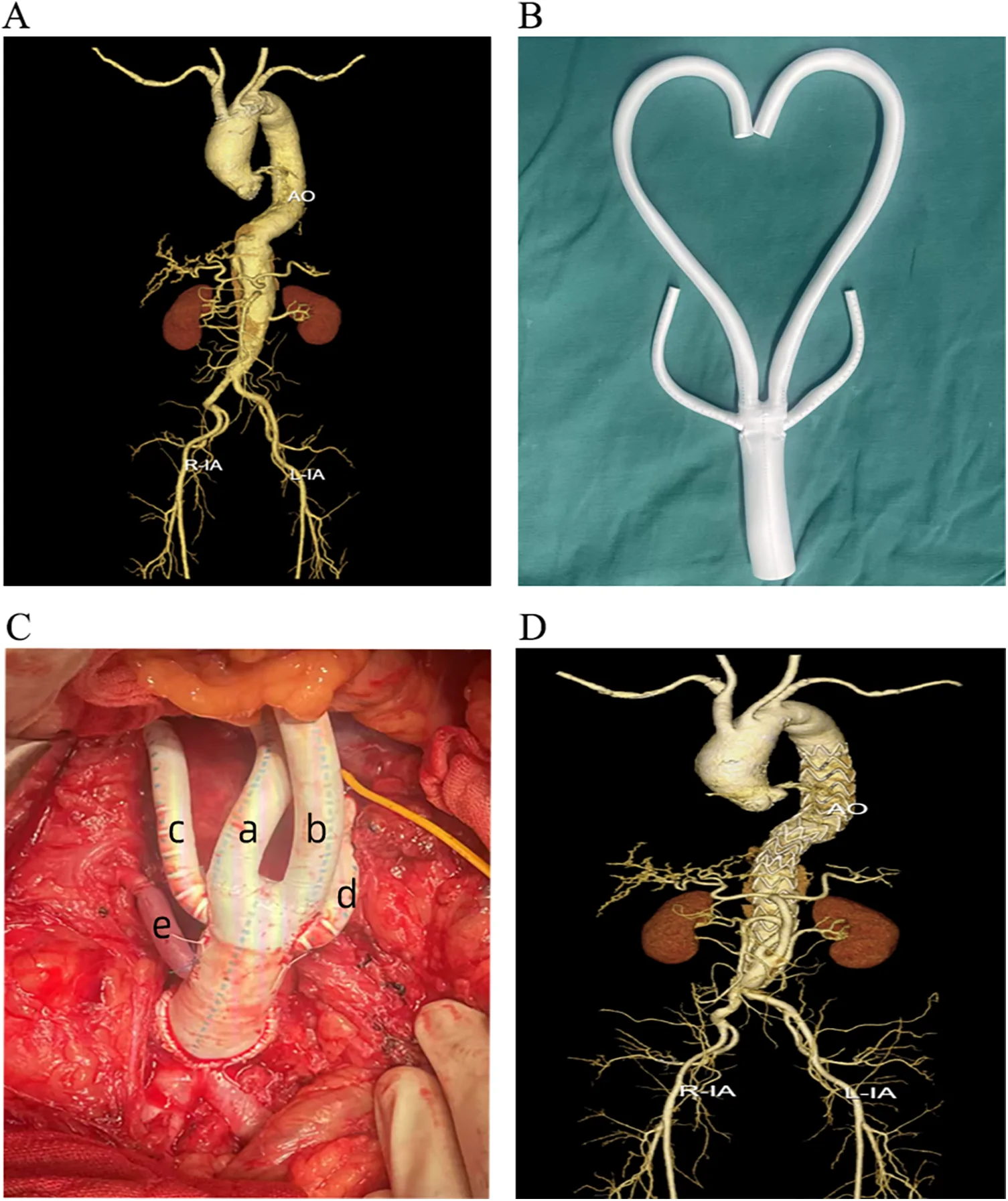
Procedural stages and intraoperative anatomical configuration of hybrid thoracoabdominal aortic aneurysm (TAAA) repair. **(A)** Preoperative three-dimensional computed tomography angiography (CTA) reconstruction: an extensive thoracoabdominal aortic aneurysm involving the visceral aortic segment. **(B)** Back-table fabrication of the custom multi-branched prosthetic graft manifold. **(C)** Intraoperative visceral artery revascularization: **a:** Superior mesenteric artery (SMA); **b:** Celiac trunk (CT); **c:** Right renal artery (RRA); **d:** Left renal artery (LRA); **e:** Right accessory renal artery (right ARA). **(D)** Postoperative three-dimensional CTA reconstruction at follow-up: complete endovascular stent-graft exclusion of the aneurysm sac and widely patent retrograde visceral and renal bypass branches.

Revascularization was performed systematically using standardized anastomotic techniques, adhering to a strict step-by-step “Inflow-First, Sequential Reperfusion” protocol in all 38 patients to maximize reproducibility and safeguard end-organ perfusion. First, inflow construction and conduit configuration were performed. When the non-aneurysmal infrarenal aorta was adequate (*n* = 27, 71.1%), a 14-mm or 16-mm straight cylindrical prosthetic graft (Gore-Tex Stretch) was anastomosed end-to-side to the anterior aortic wall under a partial tangential side-biting clamp using continuous Gore-Tex CV-3/CV-5 sutures. When the infrarenal aorta was aneurysmal, calcified, or involved by dissection (*n* = 11, 28.9%), inflow was established from the common or external iliac artery using either a straight graft or a 14 × 7 mm or 16 × 8 mm bifurcated graft (aorto-bi-iliac or ilio-iliac configuration) anastomosed end-to-side. Upon completion of the inflow anastomosis, the prosthetic trunk was flushed and de-aired, establishing an immediate, pressurized arterial inflow reservoir on the operative field.

Second, a sequential single-vessel clamping and reperfusion strategy was applied. To eliminate the catastrophic physiological stress of multi-organ simultaneous ischemia, all target visceral vessels were managed individually in strict sequence: each artery was clamped, transected or opened, anastomosed to its dedicated branch limb, and immediately reperfused before proceeding to the next vessel. No temporary vascular shunts were needed given the rapid single-vessel anastomosis times.

The standardized sequence of visceral anastomoses was as follows. Step 1: the right renal artery (RRA) was clamped and revascularized first via an end-to-end anastomosis (4-mm or 6-mm tapered branch) traversing a retrocaval infra-duodenal tunnel (median clamp time 25.0 min, IQR 20.0–30.0), and flow was immediately restored. During clamping, dedicated cold renal perfusion with chilled saline irrigation was administered to minimize renal ischemia-reperfusion injury. Step 2: the left renal artery (LRA) was clamped and revascularized second via an end-to-end anastomosis along a direct lateral retroperitoneal path (median clamp time 25.0 min, IQR 20.0–29.0). During clamping, dedicated cold renal perfusion with chilled saline irrigation was administered. Flow was immediately restored thereafter, ensuring complete restoration of bilateral renal function prior to mesenteric manipulation. Step 3: the SMA was clamped and revascularized third via an end-to-side anastomosis to the exposed SMA root along a direct anterior trajectory (median clamp time 23.0 min, IQR 16.0–25.0), and flow was immediately restored. Step 4: the celiac trunk (CT) was clamped and revascularized last via an end-to-side anastomosis to the celiac bifurcation or common hepatic artery after tunnelling through an avascular retropancreatic plane (median clamp time 20.0 min, IQR 15.0–25.0); during CT reconstruction, celiac territory collateral perfusion was continuously maintained via the newly reperfused SMA gastroduodenal and pancreaticoduodenal arcades. For supra-aortic revascularization, in patients requiring proximal stent-graft seal in Ishimaru Zone 2, a left carotid-to-subclavian artery bypass was constructed using an 8-mm ringed PTFE graft via a supraclavicular approach. After visceral artery debranching, the position of inflow was labeled with Gauze silk. Percutaneous approach of common femoral artery was established and two Proglide Perclose closure devices were pre-deployed. A 16F sheath was inserted and angiography was performed to check the patency of debranching graft and measure the length of stent graft coverage zone. Stent grafts were implanted using a conservative oversizing policy of 5%–10%, rather than >15%–20%, to minimize radial force on the fragile aortic media, and final angiography was performed to rule out endoleaks. Precise hemostasis was obtained and retroperitoneal space was closed. No drainage tube was placed.

### Optimized protocol for perioperative management

In addition to the above procedure details, our institution adopted an optimized protocol for perioperative management of TAAA, which was presented as follows:

- Normothermia with warming blanket for chest and extremities to maintain appropriate homeostasis and coagulation function.
- When feasible, preoperative CSF drainage was placed in hemodynamically stable patients who met rigorous prespecified institutional
- Volume resuscitation: maintain mean arterial pressure 90–100 mmHg
- Half-dose anticoagulation based on activated coagulation time (ACT): routine dosage for heparization is 0.5 mg/kg, maintaining 1.5-fold of baseline ACT during surgery. The anaesthesiologists remeasure ACT per hour, and add half dose of the first heparin if necessary.
- Retrograde visceral artery reconstruction (Figure 1C)
  - Grafts: GORE-TEX® Stretch Vascular Graft—Thin-wall Bifurcated and/or GORE-TEX® Stretch Vascular Graft for Vascular Access (tapered-shape 4–7 mm or 4–6 mm)
  - Sutures: GORE-TEX® Suture (CV-3 or CV-5) for inflow reconstruction and PROLENE™ Polypropylene Suture with HEMO-SEAL™ Technology for visceral outflow anastomosis.
  - Anastomosis: celiac trunk (end-to-side anastomosis to celiac bifurcation), superior mesenteric artery (end-to-side to proximal segment), bilateral renal artery (end-to-end anastomosis to proximal segment).
- Half dilation of contrast agent with normal saline in the power injector during fluoroscopy.
- Minimizing the length of stent graft coverage and the number of implanted stent grafts. Minimisation of landing zone or overlapping zone of the stent graft to 2 cm.
- Indications, Coagulation Criteria, and Timing of Preoperative CSFD
  - In this emergent/urgent cohort, preoperative prophylactic cerebrospinal fluid was used highly selectively (*n* = 2, 5.3%) following strict multidisciplinary screening, in order to balance spinal cord protection against the risk of catastrophic spinal epidural hematoma.
  - Hemodynamic criteria: Preoperative CSFD was restricted exclusively to patients with impending rupture who demonstrated absolute hemodynamic stability (mean arterial pressure ≥70 mmHg without persistent hemorrhagic shock or escalating vasopressor requirement). Preoperative CSFD was strictly contraindicated in any patient with hemodynamic instability, active hypotension, or frank free rupture.
  - Coagulation criteria: Placement required a confirmed, intact baseline coagulation profile, defined as a platelet count ≥100 × 10⁹/L, INR ≤1.2, normal activated partial thromboplastin time (aPTT), and no active coagulopathy.
  - Timing and technique relative to heparinization: CSFD catheters were placed via atraumatic lumbar puncture (L3–L4 or L4–L5) by a senior anesthesiologist prior to induction of general anesthesia and at least 60 min before any systemic heparin administration. Placement was performed under strict sterile conditions; in the event of a bloody or traumatic tap, the protocol mandated immediate catheter withdrawal and abortion of drainage. For unstable or coagulopathic patients, a “rescue CSFD” strategy was adopted, in which drainage was initiated postoperatively only upon the earliest neurological manifestation of spinal cord ischemia and after full reversal of systemic anticoagulation.

### Data collection

The prospective database of TAAA involved the following aspects:

1) Baseline information: demographics, comorbidities, onset details, Marfan syndrome, previous aortic surgeries, cardiac valve diseases, types of TAAA (dissecting, degenerative, pseudo), Crawford classification.
2) Surgical details: operation time, ASA classification, maximum diameter of TAAA, aneurysm length, inflow vessel, debranching vessels, clamping time for visceral arteries, numbers of implanted stent grafts, proximal and distal oversizing of the stent graft, bleeding volume, transfusion volume, intraoperative urine volume.

### Outcomes of interest

The primary outcome was 30-day mortality, defined as all-cause death occurring within 30 days after the index hybrid procedure, irrespective of whether the patient had been discharged. Secondary outcomes included mid- to long-term overall survival, graft patency and a free of reintervention rate. In-hospital mortality, defined as all-cause death during the index hospital stay, was also systematically documented. Other outcomes involved length of stay in ICU and postoperative hospital stay, postoperative acute kidney injury, major adverse cardiac events, pulmonary complications, bowel complications, spinal cord ischemia, and endoleaks.

### Statistical analysis

Continuous variables were tested for normality using the Shapiro–Wilk test and histogram inspection. Normally distributed variables are presented as mean ± SD and compared using independent-samples t-tests; non-normally distributed variables are presented as median (IQR) and compared using Mann–Whitney U tests. Categorical variables are presented as *n* (%) and compared using chi-square or Fisher's exact test when expected cell counts were <5.

These tests were applied to baseline characteristics, anatomical and procedural parameters, and 30-day morbidity/mortality, primarily comparing dissecting aneurysms with true aneurysms (Supplementary Tables S1, S2), with exploratory comparisons by connective tissue disease status (Marfan vs. non-Marfan). Time-to-event outcomes, including overall survival, primary visceral branch graft patency, and freedom from aortic-related reintervention, were estimated by Kaplan–Meier analysis; dissecting and degenerative subgroups were compared using the log-rank test, and hazard ratios with 95% CIs were calculated using univariate Cox proportional hazards regression.

Because follow-up was 100% complete for all primary and secondary endpoints, complete-case analysis was used throughout; missing non-mandatory secondary parameters were recorded as unavailable without imputation. All tests were two-tailed, and *P* < .05 was considered statistically significant. Analyses were using R 4.5.1.

## Results

### Patient characteristics

The cohort comprised 38 patients (31 males; median age 50.5 years, IQR 38.8–56.0). Clinical presentations were urgent impending-rupture in 37 patients (97.4%) and emergent frank rupture with shock in 1 (2.6%). Notably, 9 patients (23.7%) had Marfan syndrome, and 14 (36.8%) had prior cardiovascular interventions (Bentall procedure in 7, TEVAR in 4, valve replacement in 3). Crawford extent V (42.1%) and III (21.0%) predominated, with a mean maximum aneurysm diameter of 70.5 ± 17.8 mm. Detailed baseline characteristics, clinical symptoms, and comorbid profiles are summarized in Table 1.

**Table 1:** Baseline characteristics of 38 patients with thoracoabdominal aortic aneurysms who underwent emergent/ urgent hybrid surgery.

Label	Total(*N* = 38)
Male gender	31 (81.6%)
Age, years	50.5 (38.8, 56.0)
Body mass index, kg/m2	22.5 ± 4.3
Chest pain	20 (52.6%)
Abdominal pain	21 (55.3%)
Acute limb ischemia	1 (2.6%)
Marfan syndrome	9 (23.7%)
Coronary artery disease	3 (7.9%)
PCI	1 (2.6%)
Chronic heart failure	1 (2.6%)
Hypertension	14 (36.8%)
Arrhythmia	1 (2.6%)
Stroke	4 (10.5%)
Carotid disease	2 (5.3%)
Pulmonary disease	0 (0.0%)
Chronic kidney disease	0 (0.0%)
Diabetes mellitus	3 (7.9%)
Smoking	9 (23.7%)
CCI scores	
Mild (≤2)	26 (68.4%)
Moderate (3–4)	11 (28.9%)
Severe (≥5)	1 (2.7%)
History of valve surgery	3 (7.9%)
History of Bentall	7 (18.4%)
History of TEVAR	4 (10.5%)
History of EVAR	1 (2.6%)
Crawford classification	
I	6 (15.8%)
II	6 (15.8%)
III	8 (21.0%)
IV	2 (5.3%)
V	16 (42.1%)
Types of aneurysms	
True Aneurysm	27 (71.1%)
Pseudoaneurysm	1 (2.6%)
Aortic Dissection	10 (26.3%)
length of aneurysm (mm)	200.9 (154.0, 320.0)
The maximum diameter of the aneurysm (mm)	70.5 ± 17.8
Iliac artery aneurysm	4 (10.5%)
Follow-up, months	76. 0 (35.2, 111.8)

PCI, percutaneous coronary intervention; TEVAR, Thoracic endovascular aortic repair; EVAR, endovascular abdominal aortic repair; CCI, Charlson comorbidity index. Values are mean ± standard deviation, median (interquartile range), or number (%).

### Procedure details

The median operative time was 415 min (IQR 360–504). Single-stage hybrid repair was performed in all 38 patients. Visceral inflow was established from the infrarenal aorta in 27 patients (71.1%) and iliac arteries in 11 (28.9%). Complete four-vessel visceral debranching was accomplished in 35 patients (92.1%). In the remaining 3 patients, selective branch bypass was omitted based on favorable anatomy: three left renal arteries and one ectopic right renal artery took off from uninvolved infrarenal aorta distal to the stent-graft sealing zone and were preserved *in situ*, whereas one chronically occluded celiac trunk was adequately revascularized via retrograde collateral arcades following SMA reconstruction. A median of 3 stent-grafts were deployed for endovascular exclusion (Figure 1D). Detailed procedural metrics and branch-specific reconstruction times are presented in Table 2. Complete four-vessel visceral debranching was accomplished in 35 patients (92.1%). Incomplete four-vessel reconstruction occurred in the remaining 3 patients (7.9%). In these 3 patients, selective branch bypass was omitted based on favorable anatomy: three left renal arteries and one ectopic right renal artery arose from the uninvolved infrarenal aorta distal to the stent-graft sealing zone and were preserved *in situ*, while one chronically occluded celiac trunk was adequately perfused via retrograde collateral arcades after SMA reconstruction. Thus, the 5 non-reconstructed vessels (3 left renal, 1 right renal, and 1 celiac) were distributed across these same 3 patients, with each patient having 1 to 2 omitted vessels. Specifically, all four renal arteries, comprising three left and one right, were main renal arteries rather than accessory or polar arteries.

**Table 2:** The procedure characteristics of 38 patients with thoracoabdominal aortic aneurysms who underwent emergency/urgent hybrid surgery.

Label	Total(*N* = 38)
Surgical staging	
Single-stage	38 (100%)
Staged	0 (0%)
Inflow artery	
Abdominal aorta	27 (71.1%)
Iliac artery	11 (28.9%)
Reconstructed visceral branches per patient	4 (4, 5)
Reconstruction of SA (vessel count)	2 (5.3%)
Reconstruction of IA (vessel count)	2 (5.3%)
Reconstruction of CT (vessel count)	37 (97.4%)
Reconstruction of SMA (vessel count)	38 (100.0%)
Reconstruction of LRA (vessel count)	35 (92.1%)
Reconstruction of RRA (vessel count)	37 (97.4%)
Number of reconstructions of visceral arteries*	35 (92.1%)
Time of Inflow artery anastomosis (min)	25.0 (20.0, 30.5)
Time of CT anastomosis	20.0 (15.0, 25.0)
Time of SMA anastomosis	23.0 (16.0, 25.0)
Time of LRA anastomosis	25.0 (20.0, 29.0)
Time of RRA anastomosis	25.0 (20.0, 30.0)
Number of stent implantations	3 (2,4)
Proximal oversizing	34 (89.5%)
Distal oversizing	30 (78.9%)
Intraoperative autotransfusion utilization	21 (71.0%)
Red blood cell transfusion	12 (31.6%)
Number of lumbar drainages	2 (5.3%)
Operation time (min)	415 (360, 504)

IAA, iliac aneurysm; SA, subclavian artery; IA, iliac artery; CT, celiac trunk; SMA, Superior mesenteric artery; LRA, Left renal artery; RRA, right renal artery. Visceral arteries are defined as reconstruction of the celiac trunk, superior mesenteric artery and bilateral renal arteries. Values are mean ± standard deviation, median (interquartile range), or number (%).

### 30-day outcomes

All 38 patients required ICU admission postoperatively, with median duration of mechanical ventilation 18.0 h (IQR 10.0-24.5), ICU stay 96.0 h (IQR 46.5-203.5), and postoperative hospitalization 17 days (IQR 11-24). The 30-day mortality rate was 10.5% (*n* = 4), which was identical to the in-hospital mortality rate (10.5%, *n* = 4), as all four deaths occurred during the index hospital stay before discharge, in the impending-rupture subgroup (*n* = 37), 30-day mortality was 8.1% (3/37), the single patient with true rupture died on hemorrhagic shock due to massive hemorrhage on postoperative day (POD) 2, two from pulmonary infection-related complications (respiratory failure on POD 8 and septic shock on POD 13), and one from cerebral infarction (POD 10). Major complications included acute kidney injury in 2 patients (5.3%; one requiring CRRT with full recovery, one with residual dysfunction), pulmonary infection in 4 (10.5%; two fatal cases as above, two cured with antibiotics). Spinal cord ischemia occurred in 5 patients (13.2%), predominantly in extensive Crawford type II repairs (*n* = 3, 60.0% of SCI cases). When stratified by functional outcome, transient SCI (transient paraparesis) occurred in 3 patients (7.9%), who developed delayed lower-extremity motor and sensory weakness on postoperative days 1 to 3; all three underwent immediate rescue cerebrospinal fluid drainage (CSFD) initiated at a median of 4.5 h (IQR 2.0–8.0 h) after symptom onset, combined with hemodynamic augmentation (target MAP ≥ 90–100 mmHg) and volume optimization, and all three (100%) experienced complete or near-complete neurological recovery (Tarlov scale improvement to grade 4–5), regaining independent ambulation before hospital discharge. Permanent neurological deficits (permanent SCI) occurred in 2 patients (5.3%): one patient with Crawford type II TAAA developed immediate flaccid paraplegia and underwent emergent rescue CSFD within 2 h without neurological recovery, subsequently succumbing to extensive cerebral infarction on POD 10, while a second patient with Crawford type II TAAA exhibited severe paraparesis that achieved partial motor improvement with rescue CSFD and ultimately discharged with walker-assisted ambulation at 3-month follow-up. All 5 patients with spinal cord ischemia underwent rescue CSFD. Of these, 3 (60.0%) achieved complete or near-complete neurological recovery (Tarlov scale 4–5), 1 (20.0%) with severe paraparesis achieved partial motor improvement and was ambulating with walker assistance at 3-month follow-up, and 1 with immediate flaccid paraplegia and concurrent extensive cerebral infarction did not achieve neurological recovery and subsequently died, resulting in an overall neurological recovery rate of 80.0% (4/5) in the entire SCI cohort. No spinal hematoma, catheter-related infection, or CSFD-induced complications occurred in either the prophylactic (*n* = 2) or rescue (*n* = 5) CSFD cohorts. Gastrointestinal bleeding in 1 (2.6%; endoscopically controlled) and no myocardial infarction or hepatic failure occurred (Table 3). The outcomes of patients with dissecting TAAAs (*n* = 10) were comparable to those of the overall cohort. In this subgroup, there was one 30-day death (10.0%), attributed to cerebral infarction on postoperative day 10, and one case of spinal cord ischemia. Detailed clinical metrics for this subgroup are provided in Supplementary Table S2. The combined-risk profile is particularly relevant given the 23.7% prevalence of Marfan syndrome and other connective tissue diseases in our cohort. In these patients, fragile aortic walls predispose to late anastomotic pseudoaneurysms or patch dehiscence at debranching sites, as well as to stent-graft-induced new entries, retrograde dissection, or late sealing failure at landing zones. To mitigate these risks, several customized strategies were implemented: (1) when the infrarenal aorta was ectatic or dissected, inflow was shifted to the common or external iliac arteries (28.9% of the cohort); (2) endograft oversizing was conservatively restricted to 5%–10% rather than >15%–20%, thereby minimizing radial force on the fragile aortic media; and (3) in patients with prior prosthetic landing zones (18.4%, 7/38), the proximal endograft was securely anchored within the existing graft. Although no such catastrophic events occurred during 5-year follow-up, with a 60-month survival of 90.0% in dissecting and connective tissue cases, lifelong cross-sectional imaging surveillance remains mandatory. Among the 9 Marfan patients, there were no acute intraoperative anastomotic disruptions and no early stent-graft-induced dissections, and 30-day mortality was comparable to that of non-Marfan patients. For young, stable patients with connective tissue disease outside the acute rupture setting, open surgical repair with prosthetic aortic and visceral replacement remains the definitive gold standard.

**Table 3:** Thirty-day outcomes of 38 patients with thoracoabdominal aortic aneurysms who underwent emergency/urgent hybrid surgery.

Label	Total (*N* = 38)
Postoperative hospital stays, day	17 (11, 24)
Length of Stay in ICU, hour	96.0 (46.5, 203.5)
Duration of IMV, hour	18.0 (10.0, 24.5)
30-day mortality	4 (10.5%)
Paraplegia	5 (13.2%)
Myocardial infarction	0 (0.0%)
Stroke	1 (2.6%)
Pulmonary infection	4 (10.5%)
Gastrointestinal bleeding	1 (2.6%)
Hepatic failure	0 (0.0%)
Acute kidney failure	2 (5.3%)
Transient paraparesis	3 (7.9%)
Permanent neurological deficits	2 (5.3%)

ICU, intensive care unit; IMV, invasive mechanical ventilation. Values are median (interquartile range) or number (%).

### Long-term outcomes

The median follow-up duration was 22 months. As shown in Figure 2, the survival rates at 6, 12, 24, and 60 months postoperatively were 86.8%, 86.8%, 86.8%, and 83.0%, respectively. Four patients died within 30 days, one patient died of pneumonia-related respiratory failure at 5 months, and one patient died of cardiac tamponade due to aneurysm progression at 33 months postoperatively. The graft patency rates at 6, 12, 24, and 60 months postoperatively were 97.1%, 93.7%, 93.7%, and 93.7%, respectively. Among a total of 147 reconstructed visceral and renal branches, graft branch occlusion occurred in only two target vessels (1.4%, 2/147) during follow-up. The first was a left renal artery (LRA) branch bypass occlusion, identified on routine surveillance CTA at 3 months postoperatively (POD 92) in an asymptomatic patient; contralateral right renal graft flow was widely patent, renal function remained compensated (serum creatinine 112 µmol/L), and the patient was managed conservatively with antiplatelet therapy and serial renal function monitoring without invasive reintervention. The second was a celiac trunk (CT) branch bypass occlusion, identified on follow-up CTA at 9 months postoperatively (POD 278); the patient was completely asymptomatic with normal liver enzymes and lactate levels, owing to robust retrograde collateral perfusion from the widely patent superior mesenteric artery (SMA) via the pancreaticoduodenal arcade, and this was also managed conservatively without secondary revascularization. Notably, primary patency of the SMA bypass was 100.0% (38/38) throughout the study period, and no acute mesenteric ischemia, bowel infarction, or visceral graft-related re-laparotomy occurred. The free of reintervention rates at 6, 12, 24, and 60 months postoperatively were 97.4%, 90.5%, 90.5%, and 76.4%, respectively. One patient underwent incisional hernia repair at 8 months postoperatively, and the remaining five patients underwent coil embolization or combined stent placement for type I or type III endoleak. Similarly, long-term survival and freedom from reintervention in dissecting TAAA patients did not demonstrate noticeable divergence from non-dissecting cases (Supplementary Figure S1).

**Figure 2:**
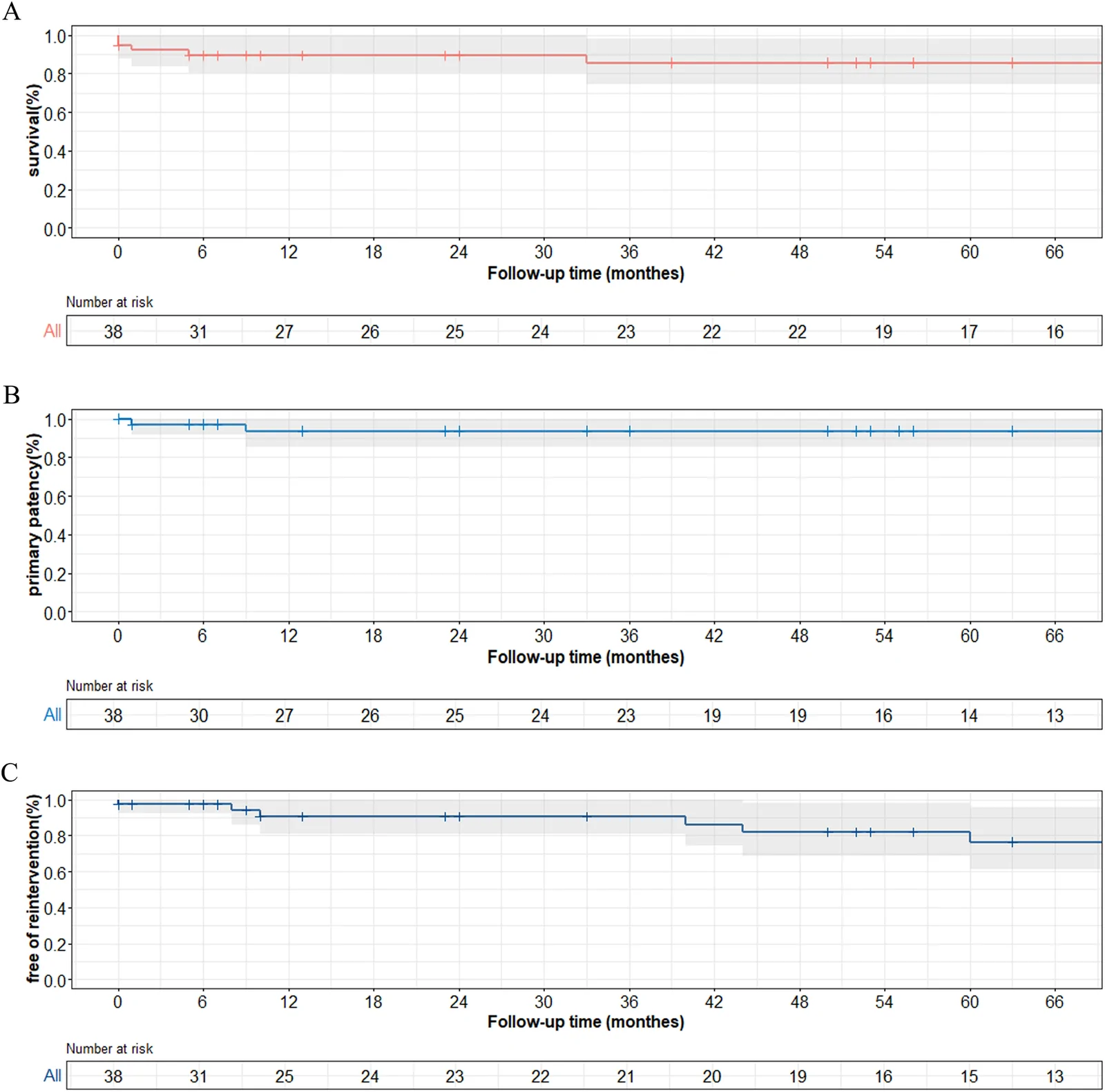
Kaplan–meier curve analysis of survival **(A)**, primary patency **(B)** and free of reintervention **(C)** of patients with thoracoabdominal aortic aneurysm treated by hybrid surgery.

## Discussion

Thoracoabdominal aortic aneurysms (TAAAs) represent life-threatening conditions, characterized by rapid progression and high mortality in cases of rupture or impending rupture. Whereas emergency open surgical repair carries significant mortality risk, hybrid repair has emerged as a viable alternative in emergent/urgent settings. This approach avoids thoracotomy and high supraceliac or descending thoracic aortic cross-clamping. Instead, it relies on low infrarenal aortic or iliac side-biting or cross-clamping to establish retrograde inflow, which substantially alleviates acute left ventricular afterload strain and minimizes profound systemic ischemia-reperfusion injury. Current evidence remains limited to case series or small single-center studies. This study presents the largest single-center experience with hybrid repair for emergent or urgent TAAA, reporting short-to-long-term outcomes.

Our cohort of 38 emergency/urgent hybrid repair patients demonstrated a median mechanical ventilation duration of 18 h, with 13.2% spinal cord ischemia   and 10.5% 30-day mortality. This compares favorably with historical emergent series, including a North American multicenter study (30-day mortality 14.4%; 1- and 5-year survival 77% and 61%), pooled estimates (12.8%) (10). and Von Meyenfeldt et al. (32%) (11), and a single-center series (26.7% overall, 36.8% emergency). However, this finding must be interpreted cautiously: 97.4% of our patients had impending rupture and were treated before free rupture, with a 30-day mortality of 8.1%, and only one patient had true rupture. The favorable outcome likely reflects multiple factors, including a younger median age (50.8 years vs. 71 years in Gkremoutis et al.'s study 9) and fewer preoperative comorbidities. Moreover, the strict exclusion of patients presenting in irreversible shock or with pre-hospital cardiac arrest inevitably enriched the cohort with individuals possessing greater physiological reserve. Although the standardized protocol ensured consistent operative execution and strict organ-protection measures, the descriptive nature of our study precludes distinguishing the protective effect of the protocol from the advantages conferred by baseline patient selection.

Mid- to long-term survival after hybrid TAAA repair remains poorly defined. The NACAAA study reported 1- and 5-year survival of 77% and 61%, with CKD stages III–V, triple or quadruple visceral reconstruction, diabetes, and chronic lung disease predicting late mortality (12). In our cohort, 92.1% underwent reconstruction of at least four visceral arteries and achieved a 5-year survival of 83.0%. These favourable outcomes potentially reflect our cohort's lower comorbidity burden compared to previously reported series, although direct comparison is limited by differences in patient selection and study design.

Hybrid TAAA repair, although immediately accessible in emergencies, entails a dual-morbidity trade-off rather than simply combining the benefits of open and endovascular techniques. It simultaneously exposes patients to the risks of both: the morbidity of laparotomy, multiple visceral anastomoses, retroperitoneal dissection, bleeding, ileus, and late incisional hernia, as well as the lifelong need for CTA surveillance, susceptibility to Type I/III endoleaks (13.2% in our cohort), component separation, and stent-graft-induced aortic injury. This intermediate compromise differs from total endovascular repair, which avoids laparotomy, and from definitive open repair, which provides durable anatomical cure.

Spinal cord ischemia remains the most debilitating and clinically significant complication following TAAA repair. In our cohort, postoperative spinal cord ischemia occurred in 13.2% of patients (5/38), representing a formidable clinical trade-off for emergent hybrid intervention. This multifactorial complication is linked to aortic clamping, hypoperfusion, and aneurysm involvement of the lumbar, subclavian, or iliac arteries. Several factors contributed to this substantial rate. First, extensive thoracoabdominal stent-graft coverage across the T8–L2 territory inevitably sacrificed critical intercostal and upper lumbar segmental arteries; notably, 3 of the 5 affected cases occurred in patients with extensive Crawford Type II TAAAs. Second, although prophylactic cerebrospinal fluid drainage is established to reduce spinal ischemia risk (13), only two hemodynamically stable patients underwent prophylactic CSFD preoperatively. One patient with postoperative paraparesis required walker-assisted ambulation by 3-month follow-up. Rescue CSFD was performed for symptomatic cases, potentially mitigating neurological deficits (14). These findings underscore that although hybrid debranching avoids high aortic cross-clamping, the risk of ischemic spinal injury appears to be within the range reported for open or total endovascular repair, mandating a proactive postoperative neuromonitoring and early rescue protocol in all emergent hybrid cases.

During follow-up, Type I or Type III endoleaks requiring secondary reintervention occurred in 13.2% of patients (5/38), comprising four Type Ia/Ib endoleaks and one late Type III endoleak. This reintervention rate falls within the range previously reported in hybrid TAAA series (9.1% to 18.9%) (9, 15–17). All five patients underwent minimally invasive endovascular secondary interventions, with complete aneurysm sac exclusion achieved in all cases without the need for open conversion. Adherence to strict proximal and distal landing zone oversizing protocols, applied in 89.5% and 78.9% of cases respectively, and were associated with a robust initial seal and acceptable mid- to long-term freedom from reintervention (76.4% at 5 years).

### Study limitations

Several limitations must be acknowledged when interpreting our findings.

First, this was a single-centre, single-arm retrospective cohort study without a historical control group from before 2013 or a contemporaneous comparator group treated with alternative strategies. Consequently, no definitive causal inference can be drawn regarding whether the standardized protocol itself directly improved perioperative survival compared with non-standardized care.

Second, given the emergent and urgent nature of TAAA presentation in this cohort, clinical data collection focused on standardized emergency triage, anatomical computed tomography parameters, and essential perioperative variables. Certain non-emergent granular parameters, such as detailed preoperative pulmonary function spirometry, baseline frailty scores, or specialized metabolic biomarkers, could not be captured retrospectively without risking missing data bias. Nevertheless, the baseline demographics, major organ comorbidities, and anatomical complexity metrics presented in Tables 1, 2 comprehensively reflect this high-risk cohort.

Third, substantial selection bias was inherent in our inclusion and triage criteria. Patients presenting with severe disturbance of consciousness, profound irreversible collapse, or those who had undergone preoperative cardiopulmonary resuscitation were deemed non-salvageable and excluded from emergent surgical intervention. This triage mechanism selected a subset of physiologically viable patients, thereby leading to an underestimation of perioperative mortality compared with unselected real-world ruptured TAAA populations.

Fourth, significant confounding stems from the distinct demographic and clinical characteristics of our cohort. The relatively young median age (50.5 years), high prevalence of Marfan syndrome (23.7%), and predominantly mild comorbidity burden (68.4% with CCI ≤ 2) reflect unique institutional referral patterns in Western China, limiting the generalizability of our mortality rates to the elderly, multi-morbid Western populations typical of other TAAA registries.

Fifth, although the median follow-up of 22 months and the 5-year Kaplan–Meier projections suggested acceptable mid-term stability, longer-term surveillance beyond 10 years is essential to fully characterize the cumulative incidence of late anastomotic aneurysms and stent-graft fatigue, particularly in the Marfan syndrome subgroup.

Finally, despite being among the larger reported single-centre series of emergent or urgent hybrid repairs, the modest sample size (*n* = 38) over a 12-year period limited the statistical power for multivariable regression modeling to identify independent predictors of adverse outcomes. Prospective, multicentre registries with comparator cohorts are warranted to substantiate the specific clinical utility of this standardized protocol.

## Conclusion

In conclusion, emergent and urgent hybrid repair represents a feasible life-saving strategy for patients with ruptured or impending-rupture complex thoracoabdominal aortic aneurysms. In this single-centre cohort managed with a standardized perioperative protocol, acceptable early mortality and durable mid- to long-term branch patency were achieved. However, the incidence of spinal cord ischemia constitutes a serious clinical concern, reflecting the inherent trade-off between rapid aortic exclusion and extensive sacrifice of intercostal arteries. This trade-off necessitates continued vigilance, standardized mean arterial pressure augmentation, and aggressive rescue cerebrospinal fluid drainage to improve neurological outcomes in acute presentations. Moreover, these outcomes should be interpreted in the context of the cohort's relatively young age and favorable baseline risk profile, and future comparative and multicentre studies are required to confirm the independent impact of this protocol on clinical outcomes.

## Data Availability

The raw data supporting the conclusions of this article will be made available by the authors, without undue reservation.

## References

[1] Tenorio ER Dias-Neto MF Lima GBB Estrera AL Oderich GS. Endovascular repair for thoracoabdominal aortic aneurysms: current status and future challenges. Ann Cardiothorac Surg. (2021) 10:744–67. 10.21037/acs-2021-taes-24 34926178 PMC8640886

[2] Isselbacher EM Preventza O Hamilton Black J Augoustides JG Beck AW Bolen MA. 2022 ACC/AHA guideline for the diagnosis and management of aortic disease: a report of the American Heart Association/American College of Cardiology joint committee on clinical practice guidelines. Circulation. (2022) 146:e334–482. 10.1161/CIR.0000000000001106 36322642 PMC9876736

[3] Böckler D Kotelis D Geisbüsch P Hyhlik-Dürr A Klemm K Von Tengg-Kobligk H. Hybrid procedures for thoracoabdominal aortic aneurysms and chronic aortic dissections—a single center experience in 28 patients. J Vasc Surg. (2008) 47:724–32. 10.1016/j.jvs.2007.12.009 18381133

[4] Girardi LN Lau C Ohmes LB Degner BC Leonard JR Abouarab A. Open repair of descending and thoracoabdominal aortic aneurysms in octogenarians. J Vasc Surg. (2018) 68:1287–96 e1283. 10.1016/j.jvs.2017.12.083 29606567

[5] Shimamura J Oshima S Ozaki K Sakurai S Hirai Y Hirokami T. Open thoracoabdominal aortic aneurysm repair: contemporary outcomes for 393 elective cases. Ann Thorac Surg. (2019) 107:1326–32. 10.1016/j.athoracsur.2018.11.021 30552886

[6] Dias-Neto M Tenorio ER Huang Y Jakimowicz T Mendes BC Kölbel T. Comparison of single- and multistage strategies during fenestrated-branched endovascular aortic repair of thoracoabdominal aortic aneurysms. J Vasc Surg. (2023) 77:1588–97 e1584. 10.1016/j.jvs.2023.01.188 36731757

[7] Dias-Neto M Vacirca A Huang Y Baghbani-Oskouei A Jakimowicz T Mendes BC. Outcomes of elective and non-elective fenestrated-branched endovascular aortic repair for treatment of thoracoabdominal aortic aneurysms. Ann Surg. (2023) 278:568–77. 10.1097/SLA.0000000000005986 37395613

[8] Chan XW Masuda Y M.T.L. Choong A Ng JJ. A systematic review and meta-analysis of total endovascular versus hybrid repair for the treatment of thoracoabdominal aortic aneurysms. J Vasc Surg. (2024) 80:1294–302 e1298. 10.1016/j.jvs.2023.11.047 38065315

[9] Gkremoutis A Schmandra T Meyn M Schmitz-Rixen T Keese M. Hybrid approach to emergent and urgent treatment of complex thoracoabdominal aortic pathology. Eur J Vasc Endovasc Surg. (2014) 48:407–13. 10.1016/j.ejvs.2014.07.010 25189829

[10] Moulakakis KG Mylonas SN Avgerinos ED Kakisis JD Brunkwall J Liapis CD. Hybrid open endovascular technique for aortic thoracoabdominal pathologies. Circulation. (2011) 124:2670–80. 10.1161/CIRCULATIONAHA.111.041582 22086877

[11] Von Meyenfeldt EM Schnater JM Reekers JA Balm R. An emergency visceral hybrid procedure for ruptured thoraco-abdominal aortic aneurysms. Eur J Vasc Endovasc Surg. (2009) 38:162–8. 10.1016/j.ejvs.2009.03.006 19398352

[12] Escobar GA Oderich GS Farber MA De Souza LR Quinones-Baldrich WJ Patel HJ. Results of the north American complex abdominal aortic debranching (NACAAD) registry. Circulation. (2022) 146:1149–58. 10.1161/CIRCULATIONAHA.120.045894 36148651

[13] Walczak S Koch R Usai MV Shehada Y Berekhoven B Austermann M. Positive impact of standardized perioperative management on spinal cord ischemia rate during endovascular repair of thoracoabdominal aneurysms. J Vasc Surg. (2025) 82:53–60. 10.1016/j.jvs.2025.02.047 40086496

[14] Spratt JR Walker KL Neal D Arnaoutakis GJ Martin TD Back MR. Rescue therapy for symptomatic spinal cord ischemia after thoracic endovascular aortic repair. J Thorac Cardiovasc Surg. (2024) 168:15–25 e11. 10.1016/j.jtcvs.2022.10.045 36509568

[15] Donas KP Czerny M Guber I Teufelsbauer H Nanobachvili J. Hybrid open-endovascular repair for thoracoabdominal aortic aneurysms: current status and level of evidence. Eur J Vasc Endovasc Surg. (2007) 34:528–33. 10.1016/j.ejvs.2007.05.023 17683957

[16] Bakoyiannis C Kalles V Economopoulos K Georgopoulos S Tsigris C Papalambros E. Hybrid procedures in the treatment of thoracoabdominal aortic aneurysms: a systematic review. J Endovasc Ther. (2009) 16:443–50. 10.1583/1545-1550-16.4.443 19702357

[17] Black SA Loftus IM Thompson MM. Staged visceral bypass with aortic relining for thoracoabdominal aneurysms: future perspectives. Perspect Vasc Surg Endovasc Ther. (2011) 23:154–60. 10.1177/1531003511411486 21810807

